# Infusing 21st Century Skill Development into the Undergraduate Curriculum: The Formation of the iBEARS Network

**DOI:** 10.1128/jmbe.00180-21

**Published:** 2021-08-31

**Authors:** Alex T. St. Louis, Penny Thompson, Tracey N. Sulak, Marty L. Harvill, Michael E. Moore

**Affiliations:** a Andrews Institute of Mathematics and Science Education, Texas Christian University, Fort Worth, Texas, USA; b College of Education and Human Sciences, Oklahoma State University, Stillwater, Oklahoma, USA; c Department of Educational Psychology, Baylor University, Waco, Texas, USA; d Department of Biology, Baylor University, Waco, Texas, USA; e STEM Education Center, University of Arkansas at Little Rock, Little Rock, Arkansas, USA

**Keywords:** 21st century skills, communication, collaboration, problem solving, project-based learning, course-based research experience, mentoring

## Abstract

The demonstrated gap between skills needed and skills learned within a college education places both undergraduates seeking gainful employment and the employers seeking highly skilled workers at a disadvantage. Recent and up-and-coming college graduates should possess 21st century skills (i.e., communication, collaboration, problem solving), skills that employers deem necessary for the workplace. Research shows that the development of this skillset can help narrow the gap in producing highly skilled graduates for the science, technology, engineering, and mathematics (STEM) workforce. We propose the development of 21st century skills by utilizing the project-based learning (PjBL) framework and creating the inclusive biologist exploring active research with students (iBEARS) program, allowing undergraduate students to hone their 21st century skills and prepare for transition and success within the workplace.

## PERSPECTIVE

College graduates entering the workplace are expected to possess sufficient content knowledge and be proficient in 21st century skills (i.e., communication, collaboration, problem solving, etc.). However, employers notice that recent graduates do not possess these necessary skill sets (1–4). We introduce a pedagogy embedded within project-based learning (PjBL) that lends itself well to 21st century skills development, within a science, technology, engineering, and mathematics (STEM) education framework. This is a departure from the traditional model found within STEM education, which is normally heavily focused on content dissemination (5), and we look to help strengthen the development of the students’ practical skills, setting a foundation of success within the global workplace.

## PROBLEM STATEMENT

Twenty-first century skills, also referred to as soft skills, represent a reconceptualization of the professional skills of the past (6, 7) steeped in a culture and workplace characterized by technological change and globalization (8). They have been defined as broad categories of skills involving thinking (e.g., creativity and innovation, critical thinking, problem solving, decision making, learning to learn), working with others (e.g., communication, collaboration/teamwork), facility with tools (e.g., information literacy, communications technology literacy), and general life skills (e.g., citizenship, life and career management, personal and social responsibility, cultural awareness) (9). Students receiving college degrees should develop a foundation of these skills for preparation in the workplace. However, studies across different global regions have shown that students enter the workforce lacking these desired skills (10, 11).

To address this problem, we propose using a project-based learning (PjBL) framework in STEM classrooms to develop 21st century skills simultaneously with science content knowledge to prepare students for careers in science and education. In this paper, we present our model for supporting university students’ 21st century skills development in the context of a biology class, through a unique PjBL experience. Under this model, university students integrate research, communication, teamwork, and problem-solving skills with content knowledge through mentoring younger learners to carry out a research project.

## 21ST CENTURY SKILLS

The value of 21st century skills has been a focus for researchers and educators seeking to prepare students for the workplace. Casner-Lotto and Barrington (11) surveyed employers about the skills employees needed to be successful in the workplace and found that employers distinguished between basic skills and applied skills. Basic skills encompassed disciplinary content knowledge, such as English grammar, science, mathematics, or second languages. Applied skills, which employers felt many new graduates lacked, included skills such as problem solving, creativity and innovation, and teamwork.

To guide educators in meeting this need, several educational organizations (e.g., International Society for Technology in Education, The Partnership for 21st Century Skills, and the MacArthur Foundation), government organizations (e.g., the European Union), and for-profit corporations have published lists of skills needed for the 21st century workplace, and the abundance of distinct yet largely overlapping lists has led researchers to synthesize 21st century skills into categories. For example, Binkley et al. (9) developed four categories of 21st century skills: “ways of thinking,” “ways of working,” “tools for working,” and “living in the world.” Ways of thinking include creativity and innovation, critical thinking, problem solving, decision making, learning to learn, and metacognition. Ways of working include communication and collaboration/teamwork, and tools for working include information literacy and communications technology literacy. Living in the world is a category encompassing local and global citizenship, life and career management, personal and social responsibility, and cultural awareness. Similarly, Kereluik et al. (8) synthesized several 21st century skills frameworks into three broad categories: “foundational knowledge” (what students need to know), “meta knowledge” (understanding how to use foundational knowledge), and “humanistic knowledge” (understanding of self and the social context).

Of the skills and competencies included in the varied frameworks of 21st century skills, employers consistently rank communication, collaboration, and critical thinking/problem solving as some of the most highly valued. In Casner-Lotto and Barrington’s (11) survey of 400 U.S. employers, the most highly ranked skills were (i) professionalism/work ethic, (ii) oral and written communication, (iii) teamwork/collaboration, and (iv) critical thinking/problem solving. In an effort to better align the skills taught in higher education institutions with the skills employers desired in college graduates, Baird and Parayitam (12) surveyed 50 employers that were active in Chamber of Commerce-sponsored job fairs in the northeastern region of the United States. These employers were asked to rate, on a five-point Likert-type scale, the importance of a list of 21st century skills the authors had compiled based on business and higher education literature. The skills that received the highest rating from these employers were interpersonal skills, critical thinking/problem solving, listening, oral communication, and professionalism. In contrast to other studies that relied on self report, Rios et al. (10) analyzed postings from careerbuilder.com and collegerecruiter.com to determine the skills most frequently requested by employers. The four most requested skills were oral communication (28%), written communication (23%), collaboration (22%), and problem solving (19%). Even among information technology professionals, soft skills such as critical thinking, problem solving, and communication were valued more than traditionally “hard” skills such as computer coding (13). To develop these skills before graduation, students need instruction and opportunities for collaboration and problem solving. PjBL creates environments where 21st century skills need to be developed and used for the project to be successful.

## PROJECT-BASED LEARNING

PjBL was developed as early as the 16th century in Europe (14), was included in Dewey’s (15) progressive education theory, and was recognized as a well-established pedagogical strategy in the latter half of the 20th century (16). PjBL is used today as “an inquiry-based instructional method that engages learners in knowledge construction by having them accomplish meaningful projects and develop real-world products” (reference 17, page 2). It is the creation of a concrete product or artifact that distinguishes PjBL from other inquiry-based pedagogical methods, such as problem-based learning (18). The focus on the creation of a tangible product means that students are focused on a shared goal and are provided some end product specifications from the instructor (19). Learning occurs as students work together to find the path toward the end product and solve problems that arise. The instructor acts as a coach, providing guidance, feedback, and suggestions as needed (19) while allowing students to lead the process. PjBL provides an environment where students not only acquire subject-matter knowledge but also gain an understanding of how that knowledge is used in authentic settings, promoting a broad understanding of the subject matter (20). PjBL has been shown to promote development of both domain-specific “hard” skills and 21st century skills such as problem solving, critical thinking, collaboration and teamwork, and lifelong learning (17). Guo et al. (17), in reviewing literature of PjBL in postsecondary/higher education, categorized prevalent cognitive, affective, and behavior outcomes (Table 1).

**TABLE 1:** Project-based learning outcomes and associated skills development based on Guo et al. (17)

PjBL outcome	Associated skill
Cognitive	Knowledge
	Conceptual understanding
	Course achievement
Cognitive strategies	Cognitive learning strategies
Affective	Perceptions of benefits (students’ perceptions of improvement of content knowledge and skills, attitude, motivation, self-efficacy)
	Perceptions of experience (attitude, satisfaction, difficulties)
Behavior	Hard skills
	Soft skills
	Engagement

## LINK BETWEEN PJBL AND 21ST CENTURY SKILLS

PjBL has been shown to support the development of 21st century skills. Bell (21) notes that the outcomes of PjBL include learning responsibility, discipline, independence, negotiation, collaboration, and communication. She also notes the use of technology for success in the 21st century, arguing for technology as a means (not an end) to help develop these skills. Bell concludes by stating that PjBL aids in helping students develop into productive members of society, where they enter a workforce built on performance outcomes and other relevant skills. Gultekin (22) shows that 21st century skills allow students to establish a foundation for real-world experiences, developing students into problem solvers and higher-order thinkers. Doppelt (23) notes an increase in the development of engagement and self-esteem. Shaw (24) explored the potential of PjBL to support 21st century skill development in high school classrooms. She used surveys and interviews to assess student and faculty perceptions of the benefits of PjBL for fostering skills such as creativity, collaboration, critical thinking, and communication. Through qualitative interviews, she found that faculty perceived collaboration, effective communication, creativity, and critical thinking as inherent attributes of the PjBL process. Quantitative survey data revealed that students perceived their skill level in these areas to be higher after taking courses consisting of at least 75% PjBL activities.

The value of PjBL to support 21st century skill development has also been shown at the university level. Musa et al. (25) used PjBL in a university-level communications class in Malaysia and surveyed students’ perceptions of the contribution of a PjBL project to their skills in teamwork, project management, communication, interpersonal relations, and problem solving. Over 70% of students agreed or strongly agreed that the PjBL experience had helped them improve their skills in each of these areas. Woodward et al. (26) integrated PjBL into two undergraduate classes for information systems majors, with the goal of developing skills in critical thinking, interpersonal communication, and teamwork, along with technical programming skills. Teams of students were assigned projects that represented realistic problems that might be found in the workplace but with the required deliverables (e.g., a working database meeting the stated specifications) clearly defined for the students by the instructors. The teams were also required to submit written documents and present their complete projects. Students then responded to surveys, at the midpoint and end of the semester, reflecting their perceptions of how the project had affected their learning of technical content and soft skills. Results showed that students reported personal growth in their soft skills during the portion of the semester when they were engaged in team-based project work. Vogler et al. (27) used student journals to gauge perceptions of 21st century skills development during a semester-long interdisciplinary PjBL experience in a university course. They found that while there were disciplinary differences in how different skills were used, all participants discussed collaboration/teamwork as a skill they were called on to use throughout the project.

## CONTEXT

**FIG 1:**
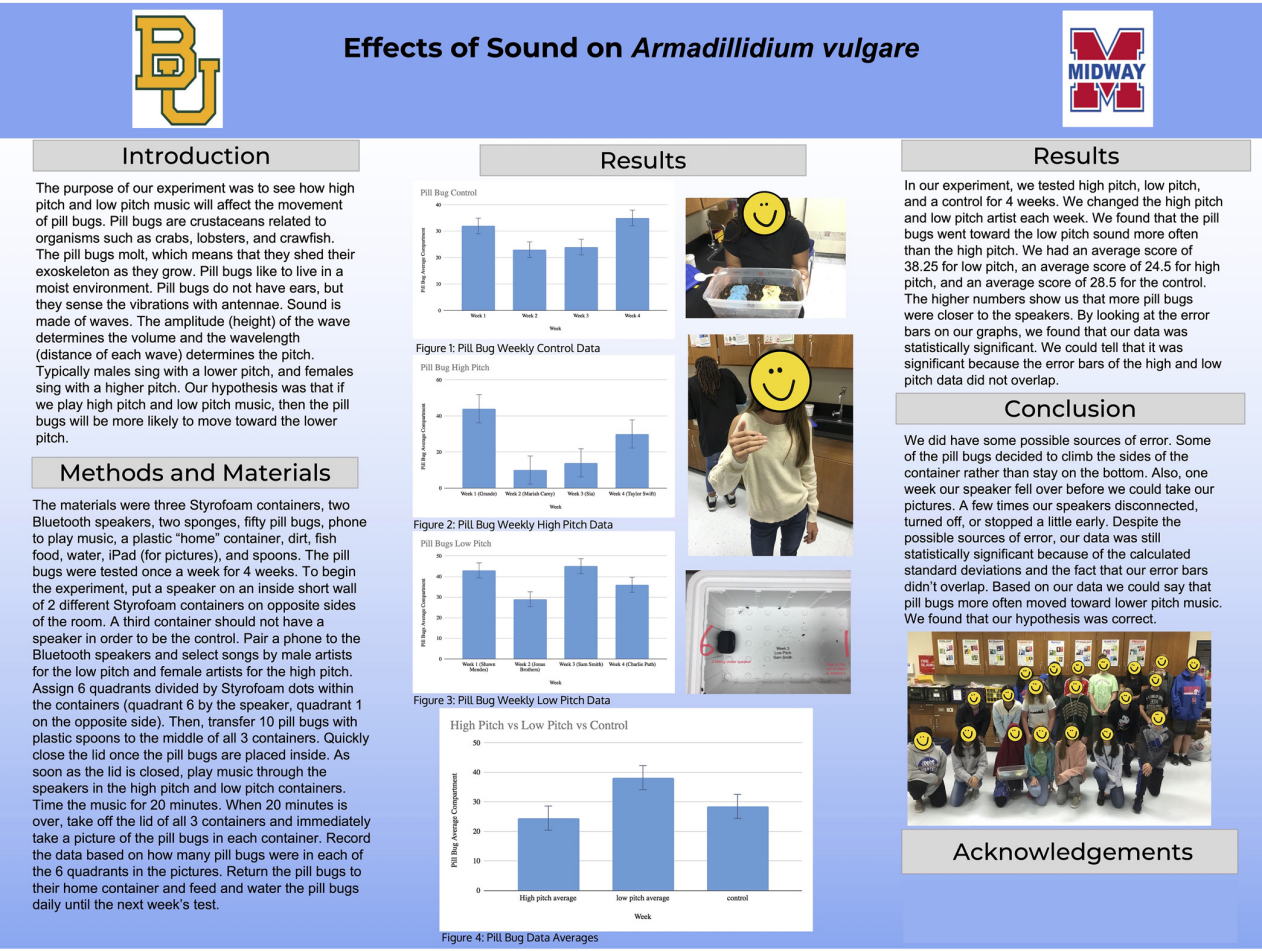
An example of one middle-level class’s collaborative research poster.

**FIG 2:**
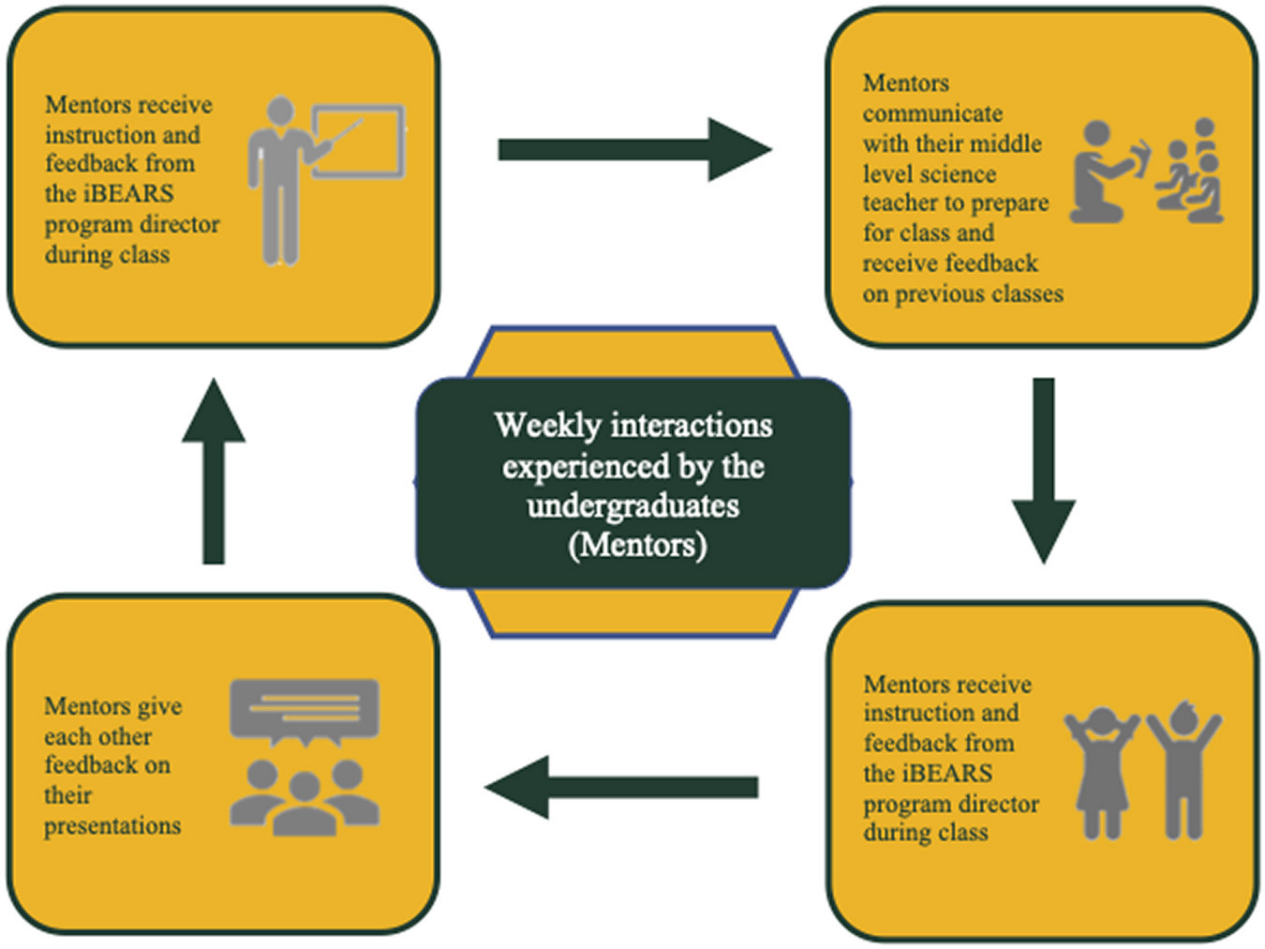
Schematic of weekly interactions that undergraduates (called mentors) experience as part of the iBEARS program.

The Inclusive Biologists Engaging in Active Research with Students (iBEARS) program was established to help include 21st century skill development into undergraduate life science curriculum utilizing PjBL while also creating mentoring and research opportunities for the up-and-coming diverse science workforce. Over the course of the semester, undergraduates work in groups of three to four students, which are assembled to be as racially and gender diverse as possible. The undergraduates’ project is to teach a class of 4th to 8th graders the scientific process through mentoring, via video conferencing, a simple research project conducted by the 4th to 8th graders. For example, one seventh-grade class measured the effects of sound on pill bugs (Fig. 1). During the project, undergraduates give weekly progress reports on each video session, create a plan of action for next week’s class, generate backup procedures, communicate (both in writing and orally) with their peers, students, teachers, and supervising instructor, divide and implement weekly tasks, and solve problems relating to technology, teaching, and experimentation (Table 2). As the project proceeds, the undergraduates begin to develop and refine their mentoring and teaching skills (Fig. 2).

**TABLE 2:** Mapping of activities to 21st century skills

Mentor activity	21st century skill(s) used
The mentor groups meet with their assigned middle-level science class using Zoom for approx 30 min once a week.	Technology/digital literacy
	Oral communication skills
In the first meeting with mentees, the mentors describe the research project and give the class a list of 10 potential organisms, mainly plants and invertebrates.	Oral communication skills
In the second meeting, the mentors help their mentees determine which independent variable the class would use for the class project.	Collaboration
	Problem solving
	Communication skills, including listening
	Decision making
In subsequent meetings, the mentors help their mentees determine materials needed for the research project and develop a supplies list that is ordered and hand-delivered to the class.	Collaboration
	Oral communication (speaking and listening)
Once the supplies are delivered, mentors, teachers, and mentees set up their research projects.	Collaboration
	Personal responsibility
Over the next several weeks, the mentors guide their mentees through the research process, asking them to explain and interpret the data they were collecting and any other observations they may have taken.	Communication (speaking and listening)
	Problem solving
	Collaboration
	Professionalism
Mentors also discuss how to write a research poster, including an introduction, methods and materials, discussion, and conclusion sections, and how to graph results.	Oral and written communication
	Technology literacy (and visual literacy)
For the remaining two weeks, mentors help incorporate the graphing and writing into a research poster template.	Written communication
	Technology literacy
Mentors and teachers establish a timeline for the class research project and create protocol for in-person classroom visits and deliveries of supplies.	Collaboration
	Professionalism

## SKILL DEVELOPMENT

To effectively develop students’ 21st century skills, the iBEARS program draws from literature on effective instruction to develop each skill. This mentor training follows recent recommendations for sustained as opposed to single session training (28). Here, we discuss effective skill development and then how we have adapted this process for the iBEARS program.

### Communication

Pairing undergraduate students with small groups of younger students has been shown to help undergraduates develop science communication skills such as understanding the audience, crafting a clear message, and explaining science concepts (29). In the iBEARS project, we accomplish this by dividing the undergraduate mentors into groups of three and assigning them a middle-level science class to mentor through a course-based research project over the semester. The final product reinforces this communication as the mentors guide their mentees through the process of making a research poster.

### Problem solving

A primary strength of PjBL is that it allows students to “work their way to the solution in their own idiosyncratic way” (reference 16, page 292) and therefore prompts the development of problem-solving skills (29). Zhong and Xu (30) assert that developing problem solving skills requires the automation of recurrent skills and the strengthening of nonrecurrent skills (i.e., generalizing). Through automating recurrent skills, which can be achieved through repetition, mental space is freed up to address nonroutine tasks. Nonrecurrent skills development can be supported by providing students with a variety of problems to navigate, which increases their adaptability, enabling them to navigate new and novel situations (30). In iBEARS, to develop nonrecurrent skills (such as generalizing and simplifying the research process so that the mentees are able to understand it), mentors watch multiple mentor groups lead their mentees through the same research procedure utilizing different organisms and research questions. Recurrent iBEARS skills (such as providing actionable feedback) are practiced weekly with the instructor’s guidance.

### Collaboration

An effective way to practice problem solving and communication skills is through group collaboration (31). Deiglmayr and Spada (32) suggest that the key to effective collaborative skill development is to support it directly through the following four steps: (i) deciding what skills to support, (ii) conceptualizing group activities to be done during the project, (iii) specifying the rules for providing adaptive support, and (iv) evaluating collaboration support. In choosing which collaborative skills to support, it is important to understand which skills are most beneficial for those who are being trained (32). To develop the collaborative skill support for iBEARS, we draw on well-known deficiencies in graduates with science degrees to develop skills in communication, collective problem solving, giving and receiving feedback, and coordinating a collaborative research project (33). Here, we provide an example of how we apply these four support steps to coordinating a collaborative research project.

**(i) Deciding what skills to support.** The success of the iBEARS project is predicated on coordinating efforts between middle-level students, middle-level science teachers, and undergraduate science students. If the mentors do not properly coordinate care for the organisms that are being studied and data collection intervals and methods, then it is highly probable that the organism will die or data will be skewed and therefore uninterpretable. Due to these factors, research project coordination is an essential collaborative skill for the undergraduate mentors to develop.

**(ii) Conceptualizing group activities.** In structuring the iBEARS project, undergraduate mentors are given one class period a week during which they review feedback from their peers, reflect on their last virtual meeting with their mentees, discuss the next steps of the research projects, and decide what needs to be communicated and sent to the middle school teacher to prepare her for the next virtual meeting. The groups receive feedback from the course instructor and can also draw on the knowledge of other mentor groups who are also preparing for their next virtual sessions.

**(iii) Specifying the rules for providing adaptive support.** Giving and receiving feedback is an important component of the iBEARS project. One of the mentor's key duties is to observe other groups’ virtual mentoring sessions and provide feedback on what went well and what could be improved. Mentors are coached on the importance of actionable feedback, and examples of exemplary feedback are discussed in class so as to provide mentors with additional guidance on how to provide effective feedback. One thing mentors are explicitly advised to think about while giving this feedback is how the collaborations could be improved (either mentor to mentor, mentor to mentee, or mentor to middle-level teacher) and how that improvement will affect the project.

**(iv) Evaluating collaboration support.** Eliciting feedback from the mentors on their experience in the iBEARS program is a process that occurs informally every week and formally at the end of every semester. The instructor solicits frequent individual and group feedback on how the collaboration is progressing, inquiring about both perceived strengths and perceived weaknesses that need to be addressed. At the end of the semester, mentors participate in group interviews where they reflect on the strengths and weaknesses of their collaborations so as to provide actionable program feedback to improve the next semester’s iBEARS project implementation.

## INSIGHTS AND FUTURE DIRECTIONS

**FIG 3:**
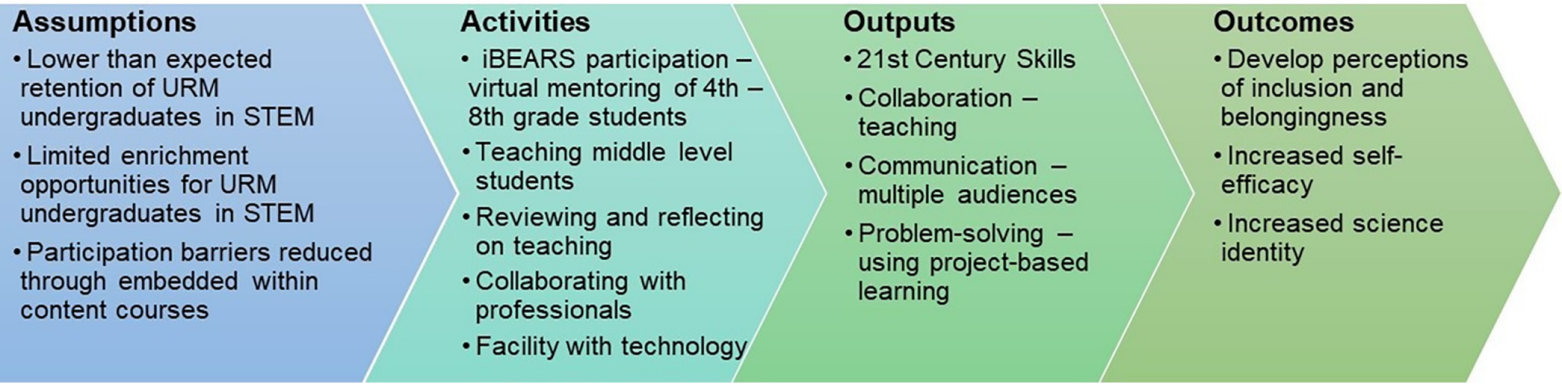
Current logic model for assessing the impact of the iBEARS program on the undergraduate mentors.

Inclusion within the science classroom is pivotal for the implementation of equity and success of students in STEM. The underlying structure of iBEARS, PjBL, allows instructors to adopt inclusive classroom practices, such as an asset-based approach to learning that creates a space for all to learn and succeed (34–36). Research has shown that utilizing inclusion within undergraduate research can increase retention rates of underrepresented students in STEM (37–40), solidify and support students’ career goals (41), and potentially compensate for inequalities these students may encounter (40, 42). There is a large body of literature that shows that underrepresented undergraduate students who actively partake in undergraduate research opportunities—including hands-on learning experiences—show an increase in measured academic success (i.e., GPA) and a positive degree graduation rate (39, 43, 44). Future research will explore the effect of participating in iBEARS to understand its effect on retention in STEM, academic success, and formation and/or completion of career goals of the middle-level science teachers (Fig. 3).

## References

[1] Hung-Lian T, Lee S, Koh S. 2000. Educational gaps as perceived by IS educators: a survey of knowledge and skill requirements. J Comput Inf Syst 41:76. doi:10.1080/08874417.2002.11646995.

[2] Jang H. 2016. Identifying 21st century STEM competencies using workplace data. J Sci Educ Technol 25:284–301. doi:10.1007/s10956-015-9593-1.

[3] Lee S, Fang X. 2008. Perception gaps about skills requirement for entry-level IS professionals between recruiters and students: an exploratory study. Inf Resour Manag J 21:39–63. doi:10.4018/irmj.2008070103.

[4] Lu MT, Chung CW, Wang P. 1998. Knowledge and skills of IS graduates: a Hong Kong perspective. J Comp Inf Syst 39:40–47. doi:10.1080/08874417.1999.11647388.

[5] Henderson C, Finkelstein N, Beach A. 2010. Beyond dissemination in college science teaching: an introduction to four core change strategies. J Coll Sci Teach 39:18–25.

[6] De La Harpe B, Radloff A, Wyber J. 2000. Quality and generic (professional) skills. Qual High 6:231–243. doi:10.1080/13538320020005972.

[7] Shuman LJ, Besterfield-Sacre M, McGourty J. 2005. The ABET ‘professional skills’—Can they be taught? Can they be assessed? J Engineering Education 94:41–55. doi:10.1002/j.2168-9830.2005.tb00828.x.

[8] Kereluik K, Mishra P, Fahnoe C, Terry L. 2013. What knowledge is of most worth: teacher knowledge for 21st century learning. J Digit Learn Teach Educ 29:127–139. doi:10.1080/21532974.2013.10784716.

[9] Binkley M, Erstad O, Herman J, Raizen S, Ripley M, Miller-Ricci M, Rumble M. 2012. Defining twenty-first century skills, p 17–66. *In* P Griffin, B McGaw, & E Care (ed), Assessment and teaching of 21st century skills. Springer, Dordrecht. doi:10.1007/978-94-007-2324-5_2.

[10] Rios JA, Ling G, Pugh R, Becker D, Bacall A. 2020. Identifying critical 21st-century skills for workplace success: a content analysis of job advertisements. Educ Res 49:80–89. doi:10.3102/0013189X19890600.

[11] Casner-Lotto J, Barrington L. 2006. Are they really ready to work? Employers' perspectives on the basic knowledge and applied skills of new entrants to the 21st century US workforce. https://files.eric.ed.gov/fulltext/ED519465.pdf.

[12] Baird AM, Parayitam S. 2019. Employers’ ratings of importance of skill and competencies college graduates need to get hired. Educ Train 61:622–634. doi:10.1108/ET-12-2018-0250.

[13] Bailey JL, Stefaniak G. 2001. Industry perceptions of the knowledge, skills, and abilities needed by computer programmers, p 93–99. *In* Proceedings of the 2001 ACM SIGCPR conference on computer personnel research. Association for Computing Machinery. doi:10.1145/371209.371221.

[14] Hugg R, Wurdinger S. 2007. A practical and progressive pedagogy for project based service learning. Int J Teaching Learn High Educ 19:191–204.

[15] Dewey J. 1900. The school and society. University of Chicago Press. Chicago, IL.

[16] Adderley K. 1975. Project methods in higher education. Society for Research into Higher Education. London, UK.

[17] Guo P, Saab N, Post LS, Admiraal W. 2020. A review of project-based learning in higher education: student outcomes and measures. Int J Educ Res 102:101586. doi:10.1016/j.ijer.2020.101586.

[18] Helle L, Tynjala P, Olkinuora E. 2006. Project-based learning in post-secondary education—theory, practice, and rubber sling shots. High Educ 51:287–314. doi:10.1007/s10734-004-6386-5.

[19] Savery JR. 2006. Overview of problem-based learning: definitions and distinctions. Interdiscip J Probl Based Learn 1:9–20. doi:10.7771/1541-5015.1002.

[20] Blumenfeld PC, Soloway E, Marx RW, Krajcik JS, Guzdial M, Palincsar A. 1991. Motivating project-based learning: sustaining the doing, supporting the learning. Educ Psychologist 26:369–398. doi:10.1080/00461520.1991.9653139.

[21] Bell S. 2010. Project-based learning for the 21st century: skills for the future. Clearing House 83:39–43. doi:10.1080/00098650903505415.

[22] Gultekin M. 2005. The effect of project based learning on learning outcomes in the 5th grade social studies course in primary education. Educ Sci Theory Pract 5:548–556.

[23] Doppelt Y. 2003. Implementing and assessment of PBL in a flexible environment. Int J Technol Des Educ 13:255–272. doi:10.1023/A:1026125427344.

[24] Shaw RL. 2018. Using project-based learning to cultivate 21st century skills in STEM education. PhD thesis. Lamar University, Beaumont, Texas.

[25] Musa F, Mufti N, Latiff RA, Amin MM. 2012. Project-based learning (PjBL): inculcating softskills in 21st century workplace. Procedia Soc Behav Sci 59:565–573. doi:10.1016/j.sbspro.2012.09.315.

[26] Woodward BS, Sendall P, Ceccucci W. 2010. Integrating soft skill competencies through project-based learning across the information systems curriculum. J Inf Syst Educ 8.

[27] Vogler JS, Thompson P, Davis DW, Mayfield BE, Finley PM, Yasseri D. 2018. The hard work of soft skills: augmenting the project-based learning experience with interdisciplinary teamwork. Instr Sci 46:457–488. doi:10.1007/s11251-017-9438-9.

[28] Moore ME, Naganathanm A, Blummer SL, Goller CC, Misra A, Raut SA, Swammy U, Wick S, Wolyniak MJ. 1975. Facilitating long-term mentoring to effectively implement active learning instruction: the formation of the promoting active learning and mentoring (PALM) network. Biochem Pharmacol 24:1639–1641. doi:10.1128/jmbe.v21i3.2203. 33294101 PMC7669288

[29] Grant BL, Liu X, Gardella JA. 2015. Supporting the development of science communication skills in STEM university students: understanding their learning experiences as they work in middle and high school classrooms. Int J Sci Educ B Commun Public Engagem 5:139–160. doi:10.1080/21548455.2013.872313.

[30] Zhong L, Xu X. 2019. Developing real life problem-solving skills through situational design: a pilot study. Education Tech Res Dev 67:1529–1545. doi:10.1007/s11423-019-09691-2.

[31] Lai E. 2011. Collaboration: a literature review. Pearson Publisher.

[32] Deiglmayr A, Spada H. 2010. Collaborative problem-solving with distributed information: the role of inferences from interdependent information. Group Process Intergroup Relat 13:361–378. doi:10.1177/1368430209342259.

[33] American association for the advancement of science. 2010. Vision and change: a call to action. AAAS, Washington, DC. https://visionandchange.org/.

[34] Forrester G, Kurth J, Vincent P, Oliver M. 2020. Schools as community assets: an exploration of the merits of an asset-based community development (ABCD) approach. Educ Rev 72:443–458. doi:10.1080/00131911.2018.1529655.

[35] Cho A, Herrera RG, Chaidez L, Uriostegui A. 2019. The “Comadre” project: an asset-based design approach to connecting low-income Latinx families to out-of-school learning opportunities, p 1–14. *In* Proceedings of the 2019 CHI conference on human factors in computing systems.

[36] Shah RW, Troester JMS, Brooke R, Gatti L, Thomas SL, Masterson J. 2018. Fostering eABCD: asset-based community development in digital service-learning. J High Educ Outreach Engagem 22:189–222.

[37] Cole D, Espinoza A. 2008. Examining the academic success of Latino students in science technology engineering and mathematics (STEM) majors. J Coll Stud Dev 49:285–300. doi:10.1353/csd.0.0018.

[38] Hurtado S, Eagan MK, Cabrera NL, Lin MH, Park J, Lopez M. 2008. Training future scientists: predicting first-year minority student participation in health science research. Res High Educ 49:126–152. doi:10.1007/s11162-007-9068-1. 23503996 PMC3596162

[39] Jones MT, Barlow AEL, Villarejo M. 2010. Importance of undergraduate research for minority persistence and achievement in biology. J High Educ 81:82–115. doi:10.1080/00221546.2010.11778971.

[40] Finley A, McNair T. 2013. Assessing underserved students’ engagement in high-impact practices. Association of American Colleges and Universities, Washington, DC.

[41] Schultz PW, Hernandez PR, Woodcock A, Estrada M, Chance RC, Aguilar M, Serpe RT. 1975. Patching the pipeline: reducing educational disparities in the sciences through minority training programs. Educ Eval Policy Anal 33. doi:10.3102/0162373710392371. PMC3839574 24285910

[42] Kinzie J, Gonyea R, Shoup R, Kuh GD. 2008. Promoting persistence and success of underrepresented students: lessons for teaching and learning. New Dir Teach Learn 115:21–38. doi:10.1002/tl.323.

[43] Russell SH, Hancock MP, McCullough J. 2007. Benefits of undergraduate research experiences. Science 316:548–549. doi:10.1126/science.1140384. 17463273

[44] Crawford B. 2008. Creating effective undergraduate research programs in science: the transformation from student to scientist. Sci Educ 9:583–585. doi:10.1002/sce.20336.

